# Turning value into action: Healthcare workers using digital media advocacy to drive change

**DOI:** 10.1371/journal.pone.0250875

**Published:** 2021-04-29

**Authors:** Marissa A. Boeck, Catherine J. Juillard, Rochelle A. Dicker, Bellal A. Joseph, Joseph V. Sakran

**Affiliations:** 1 Department of Surgery, Zuckerberg San Francisco General Hospital, University of California, San Francisco, San Francisco, CA, United States of America; 2 Department of Surgery, University of California, Los Angeles, Los Angeles, CA, United States of America; 3 Department of Surgery, University of Arizona College of Medicine, Tucson, AZ, United States of America; 4 Department of Surgery, Johns Hopkins Hospital, Baltimore, MD, United States of America; Stevens Institute of Technology, UNITED STATES

## Abstract

**Background:**

The standard method of sharing information in academia is the scientific journal. Yet health advocacy requires alternative methods to reach key stakeholders to drive change. The purpose of this study was to analyze the impact of social media and public narrative for advocacy in matters of firearm-related injury and death.

**Study design:**

The movement *This Is Our Lane* was evaluated through the #ThisIsOurLane and #ThisIsMyLane hashtags. Sources were assessed from November 2018 through March 2019. Analyses specifically examined message volume, time course, global engagement, and content across Twitter, scientific literature, and mass media. Twitter data were analyzed via Symplur Signals. Scientific literature reviews were performed using PubMed, EMBASE, Web of Science, and Google Scholar. Mass media was compiled using Access World News/Newsbank, Newspaper Source, and Google.

**Results:**

A total of 507,813 tweets were shared using #ThisIsOurLane, #ThisIsMyLane, or both (co-occurrence 21–39%). Fifteen scientific items and n = 358 mass media publications were published during the study period; the latter included articles, blogs, television interviews, petitions, press releases, and audio interviews/podcasts. Peak messaging appeared first on Twitter on November 10^th^, followed by mass media on November 12^th^ and 20^th^, and scientific publications during December.

**Conclusions:**

Social media enables clinicians to quickly disseminate information about a complex public health issue like firearms to the mainstream media, scientific community, and general public alike. Humanized data resonates with people and has the ability to transcend the barriers of language, culture, and geography. Showing society the reality of caring for firearm-related injuries through healthcare worker stories via digital media appears to be effective in shaping the public agenda and influencing real-world events.

## Introduction

Firearm-related injury and death in America is a public health crisis. Every day one hundred Americans are killed with guns, not to mention the many more who survive with life-altering disabilities [1]. Estimates indicate firearms were the most common cause of violence-related death in the United States (U.S.) in 2017 [2]. Like earlier public health epidemics caused by motor vehicles and tobacco, firearms require a standardized method of study to both quantify the magnitude of the problem and qualify risk factors in order to develop effective prevention and mitigation tactics [3].

The American College of Physicians (ACP) e-published a position paper on firearm injuries and deaths on October 30^th^, 2018 [4]. This new publication expanded on the organization’s comprehensive 2014 policy recommendations, which led to minimal action during its five years of existence despite being endorsed by 52 multi-disciplinary groups. The recent ACP position paper prompted the National Rifle Association of America (NRA) to respond on November 7^th^, 2018 with a message on Twitter saying, “Someone should tell self-important anti-gun doctors to stay in their lane” [5]. Occurring at a time primed with escalating episodes of firearm violence and efforts to limit physician-questioning of firearm safety practices in the home [6], this tweet sparked a global response from incensed healthcare professionals. Ranging from everyday stories to graphic photos about caring for firearm-injured patients, the public caught a glimpse of the daily tragedies seen by the medical community, and were able to hear the raw experiences of firearm violence survivors and their families.

Public narrative, a leadership technique described by Marshall Ganz, involves transforming values into action and argues that it is not only important that people speak, but what, how, and why they say it matters [7]. Historically, the standard method of sharing information in academic medicine and public health has been through peer-reviewed scientific publications. However, the average paper in a standard journal is cited less than once in the two years following its publication [8]. While visual abstracts on social media hold promise to magnify these publications to reach bigger audiences [9], a gap still exists in disseminating data-driven information to key stakeholders. While traditional media may not tell people what to think, it does tell them what to think *about*, effectively setting the public agenda on an issue [10, 11]. It follows that media advocacy is the “strategic use of mass media to apply pressure to advance healthy public policy” [10], amplifying authentic voices for policymakers to hear and shifting the power back to the community. Studies have looked at the impact of media for public health advocacy, activism, and health policy change for tobacco, alcohol, road safety, and maternal and newborn health [10, 12], the inclusion of stories to more effectively communicate with policymakers [13], and the use of social media to facilitate activism and civic engagement [14–16]. Yet the power of social media for healthcare advocacy and health policy change is not well understood [12].

The purpose of this study was to analyze the impact of social media, and the role of public narrative for advocacy, as it relates to firearm-related injury and death. We hypothesized that social media would have a larger message volume, earlier onset, broader global participation, more variable content, and would likely potentiate traditional information streams, providing a tipping point for action and subsequent real-world events. We also explored possible temporal associations between publication volume and firearm violence incidents, organized events, and firearm policy actions, hypothesizing there would be increased message activity surrounding significant events.

## Materials and methods

### Data collection

All of the data in this study are publicly available, and therefore not subject to ethical review and approval. Twitter data were retrieved via Symplur Signals (Symplur LLC, California, USA), a healthcare social media analytics platform. Symplur Signals access was obtained for one-month, during which time 19 weeks of Twitter data were available for the hashtags #ThisIsOurLane and #ThisIsMyLane, from November 7^th^, 2018 (the date of the NRA tweet) to March 15^th^, 2019. The hashtag #ThisIsOurLane was introduced on Twitter on November 7^th^, 2018 and registered with Symplur on November 8^th^, 2018; #ThisIsMyLane was introduced on November 8^th^, 2018 and registered on November 10^th^, 2018. Symplur retrieves hashtag data from Twitter seven days prior to hashtag registration, up to a maximum of 2000 tweets per day (company email correspondence). The account @ThisIsOurLane was created on November 10^th^, 2018, aiming to represent medical professionals who care for victims of firearm violence, and proposing effective solutions to prevent firearm injuries and deaths. Account activity is not routinely tracked by Symplur, so formal, in-depth analytic data were not available (company email correspondence).

Scientific publications and mass media items were queried using the terms #ThisIsOurLane, #ThisIsMyLane, “this is our lane,” “this is my lane,” with or without “gun violence,” and were limited to those items published from November 7^th^, 2018 to March 15^th^, 2019 to align with Symplur Twitter data availability. Scientific publications were defined as those items appearing in or associated with an academic journal or association, and were compiled using PubMed, EMBASE, Web of Science, and Google Scholar. These publications included peer-reviewed studies, opinion pieces, sponsored events, podcasts, petitions, graphics, or news articles. Mass media items included mainstream media publications, television or radio programs, and general websites excluding Twitter, and were accessed using Access World News/Newsbank, Newspaper Source, and Google. For the latter, search results in the form of URLs (uniform resource locator) were exported to a spreadsheet using the plugin SEOquake (Semrush Inc, Trevose, PA, USA).

U.S. mass shooting events were identified using the Gun Violence Archive database. This group defines a mass shooting as any event with “four or more victims injured or killed excluding the subject/suspect/perpetrator at one location” [17]. Significant firearm-related events (i.e. mass shootings, organized events, and/or policy actions) were identified either by volume spikes on Twitter, repeated mentions in scientific publications and/or mass media items, and/or author identification, with subsequent content review and author consensus for inclusion.

### Data analysis

Symplur Signals data were assessed separately for each hashtag, as the hashtags could co-occur in the same message. Tweets refer to the number of unique message posts on Twitter, and participants or users are the number of distinct accounts that have sent a tweet at least once. Impressions are calculated as the sum of the number of times an account has tweeted multiplied by the number of an account’s followers, repeated for all accounts; these reflect the theoretical maximum number of Twitter users a tweet could directly reach in a follower’s timeline [18]. To create the healthcare stakeholder categories, Symplur uses a combination of self-identification information in public Twitter biographies and algorithms, machine learning models, and manual human evaluation. Users are divided into default categories, including doctor, healthcare professional (i.e. nurses, pharmacists, respiratory therapists etc.), researcher/academic, and various types of organizations, among others [19]. Tweet content was assessed by reviewing the 25 most popular tweets based on retweets for #ThisIsOurLane.

Scientific publication and mass media item searches were individually reviewed for relevance to #ThisIsOurLane and #ThisIsMyLane. Inclusion criteria were publication between November 7^th^, 2018 and March 15^th^, 2019, and a reference to the advocacy movement via the use of one or both hashtags and/or phrases in the context of firearms. Unrelated items were removed and qualifying documents were compiled into an Excel spreadsheet. Information collected included the search source, publisher, type of item, publishing medium, title, author, date, URL, location, language, Altmetric score (if applicable), and notes. Exact duplicates were removed. Mass media items with the same author and title but different date, publisher, and/or publishing medium (e.g. internet versus newspaper) were included. Items were sorted by date (if available) and grouped by type of item (article, audio interview/podcast, video, petition, blog, event, fundraiser, letter, graphic, press release, summary, transcript, or testimony). Location and language were determined via the publisher/host website. Altmetric Attention Scores were collected for scientific articles via the journal’s website, when available. This publicly available score is derived from an automated, weighted algorithm sourced from the Web, and is an indicator of the amount of attention a research item has received across various social and traditional media, blogs, and reference manager platforms. Although the scores are dependent on the context and not normalized, in general an article scoring 20 or higher is receiving more attention than most of its contemporaries, a time period comprised of three months surrounding the article’s publication date [20].

## Results

### Social media (Twitter)

**Fig 1:**
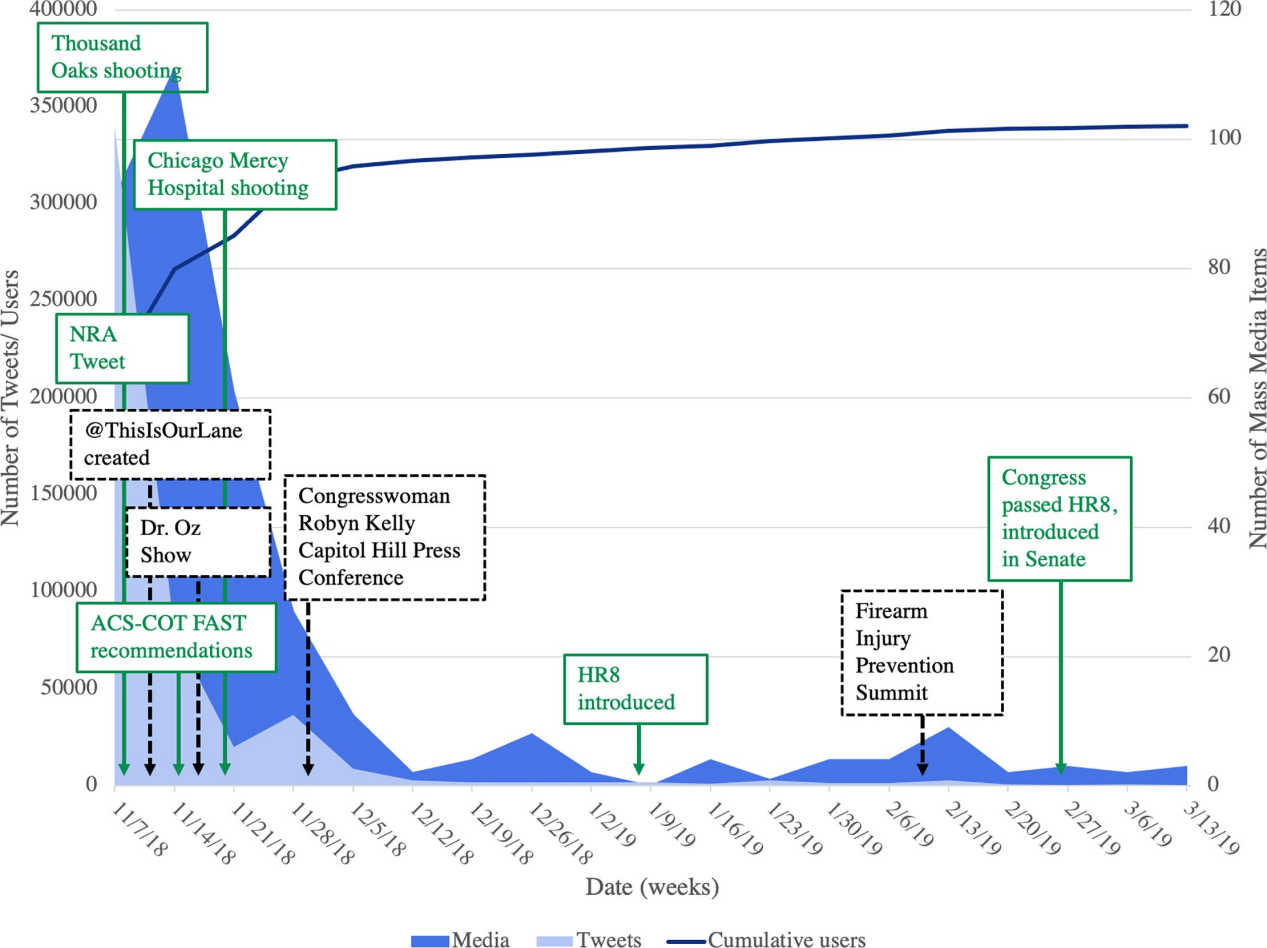
Twitter versus mass media volume and cumulative users on Twitter by week.

**Fig 2:**
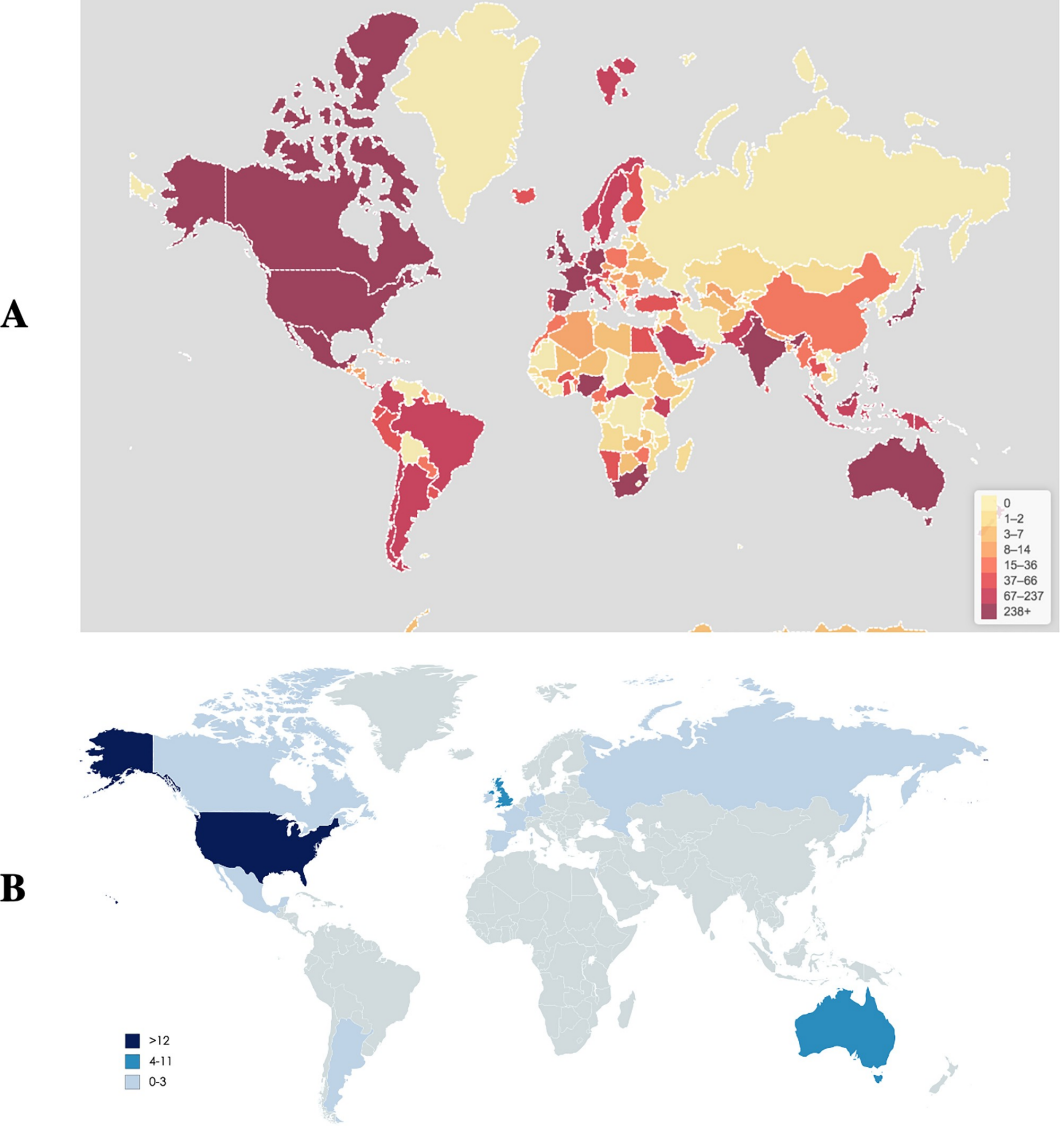
World map of #ThisIsMyLane Twitter users by country (A) and Mass media item locations (B). Scales refer to (A) number of users on Twitter (B) number of mass media items. (A) Symplur Signals, a healthcare social media analytics platform. (B) Republished from Mapchart.net under a CC BY license, with permission from Mapchart.net, original copyright 2020.

Over the 19-week study period, 507,813 tweets were posted using either #ThisIsOurLane, #ThisIsMyLane, or both, with over 1.2 billion impressions between the two hashtags. Many tweets used multiple hashtags, with #ThisIsOurLane and #ThisIsMyLane co-occurring 21–39% of the time. The most tweets were sent on November 10^th^, 2018 for both hashtags (#ThisIsOurlane n = 34,760, #ThisIsMyLane n = 114,921), with additional spikes on November 20^th^, 2018 for #ThisIsOurLane (n = 10,193) and November 29^th^, 2018 for #ThisIsMyLane (n = 18,214) (Fig 1). User profiles varied between the two hashtags; most using #ThisIsMyLane were unknown due to a lack of user-provided data, while users of #ThisIsOurLane mainly identified as doctors, researchers/academics, and journalists/media. For both hashtags, many participants were located in the United States and most were tweeting in English. Overall, users participated from over 200 countries in 26 languages (Fig 2A). For tweets with media, most of the photos shared were graphic, showing blood-soaked scrubs or surgical tools, real gunshot wounds to organs, or empty trauma bays covered in blood, accompanied by everyday stories of the devastation bullets cause. Regarding the 25 most popular tweets for #ThisIsOurLane based on retweets (range n = 713–17,600), 76% were from doctors, 56% included media (photo or video), and 16% tagged other users. Further information on hashtag users and demographics is provided in Table 1.

**Table 1:** Twitter hashtag & user statistics.

Characteristic		#ThisIsOurLane N (%)	#ThisIsMyLane N (%)
Total tweets		182,120 (100)	325,693 (100)
Tweets with mentions		174,527 (96)	320,788 (98)
Retweets		166,422 (91)	315,569 (97)
Tweets with media		79,069 (43)	136,349 (42)
Tweets with links		36,755 (20)	15,167 (5)
Impressions^a^		582,093,241	703,687,561
Tweets per hour		59.3	106
Median tweets per user		1	1
Users who made 1 tweet		63,684 (71)	107,064 (68)
Users who made 10+ tweets		1,681 (2)	3,280 (2)
Users		89,758	158,234
**Influencers by Tweets**^b^	Doctor	(45)	(10)
	Researcher/Academic	(4)	--
	Journalist/media	(4)	--
	Individual other health	(4)	--
	Individual non-health	(4)	(7)
	Caregiver/advocate	(3)	--
	Organization advocate/Support	(3)	--
	Healthcare provider	(3)	(2)
	Patient	(2)	(2)
	Organization other healthcare	(1)	--
	Unknown	(28)	(79)
**Locations**^c^	United States	37,555 (42)	66,185 (42)
	Canada	2,986 (3)	4,519 (3)
	United Kingdom	1,655 (2)	4,064 (3)
	Australia	1,290 (1)	2,272 (1)
	France	624 (1)	671 (0.4)
**Languages**^b^	English	172,624 (95)	316,518 (97)
	Korean	4,633 (2)	173 (0.05)
	Spanish	936 (0.5)	2,655 (1)
	French	1,371 (1)	1,528 (0.5)
	German	624 (0.3)	1,548 (0.5)
	Japanese	769 (0.4)	872 (0.3)

^a^Impressions: Theoretical maximum number of Twitter users a tweet could directly reach in a follower’s timeline

^b^N (%) of total tweets

^c^Based on free-form data entered into Twitter profiles, N (%) of total users, unidentifiable locations: 40,544 (TIOL, 45%), 69,965 (TIML, 44%)

Another metric of the movement on Twitter was the @ThisIsOurLane account. Within days, the account’s followers grew exponentially, reaching nearly 32,000 followers at the time of this publication through n = 3,189 tweets [21].

### Mass media

**Fig 3:**
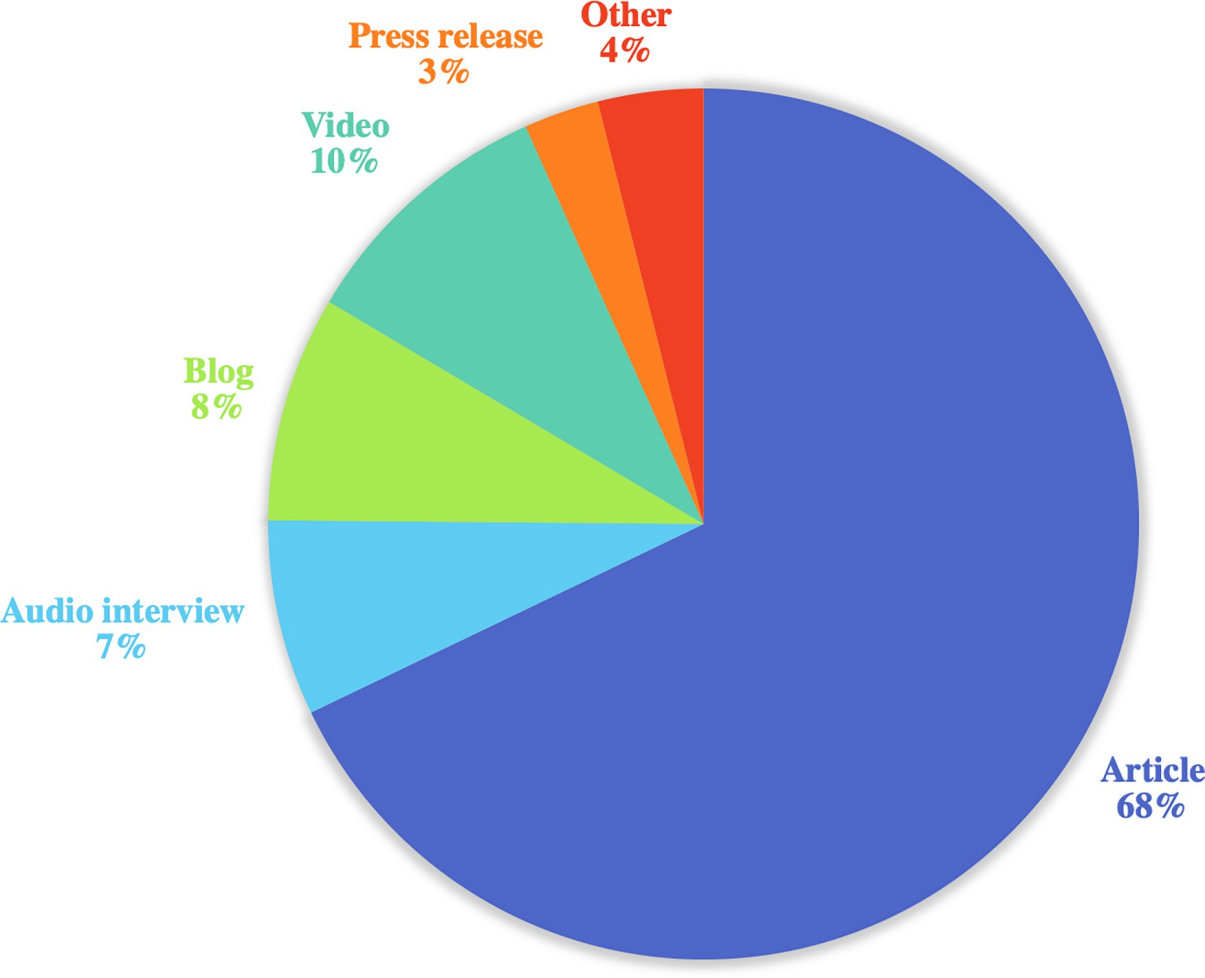
Mass media volume by type.

Out of n = 624 results across various search engines, n = 358 met study inclusion criteria. Mass media items were first published on November 8^th^, 2018 (n = 3), which included articles on Fox 2 Now St. Louis [22], The Daily Dot [23], and The Daily Beast [24]. During the 133-day study period the highest number of pieces circulated on both November 12^th^, 2018 and November 20^th^ (n = 32, 9% each) (Fig 1). The most common type of publication was articles (n = 243, 73%), followed by videos (n = 35, 10%), blogs (n = 30, 8%), and audio interviews/podcasts (n = 26, 7%) (Table 2 and Fig 3). Most were from American publishers/hosts (n = 314, 88%) (Fig 2B) written in English (n = 345, 96%) or Spanish (n = 11, 3%).

**Table 2:** Scientific literature & mass media items.

Category	Type	Number (%)	Publishers/Hosts	Locations
Scientific N = 15	Article	10 (67)	Annals of Internal Medicine, NEJM, JAMA Network Open, JAMA, Science, Academic Emergency Medicine, BMJ, Ann Internal Medicine, Open Forum Infect. Disease	United States, United Kingdom
	Event	2 (13)	Asian Pacific American Medical Students, UCLA David Geffen School of Medicine	United States
	Graphic	1 (7)	Annals of Internal Medicine	United States
	Podcast	1 (7)	JAMA Medical News	United States
	Petition	1 (7)	Annals of Internal Medicine	United States
Mass media N = 358	Article^a^	243 (68)	The Daily Dot, The Daily Beast, Medscape, Newsweek, Mashable, USA Today, Chicago Tribune, Daily Mail, Forbes, Physician’s Weekly, BBC News, Vox, ABC News, The Washington Times, The Philadelphia Inquirer, People, The Baltimore Sun, The Evening Standard, SF Weekly, The Hill, NY Post, San Francisco Chronicle, The Washington Post, The Daily Telegraph, Los Angeles Times, Slate, The Times, The Globe and Mail, The New York Times, The Independent, Time, The Guardian, BuzzFeed, Associated Press, Chicago Sun Times, Politico, Huffington Post	United States, United Kingdom, Australia, Unknown, Spain, Canada, Singapore, Mexico, Argentina, Israel, France, Ireland
	Video^a^	35 (10)	CNN, NBC, CBS, PBS, ABC, CSPAN, Medscape, Doctor Oz, Huffington Post, Yahoo, USA Today, NowThis	United States, Canada, Germany, Australia, Soviet Union
	Blog^a^	30 (8)	Doximity, Medium, JournalFeed, ACP Advocate Blog, Emergency Medicine News, KevinMD, Health Affairs, The Mighty, Brady Campaign, Wellness Rounds	United States, Unknown
	Audio interview/podcast^a^	26 (7)	NPR & affiliates (Fresh Air, All Things Considered, The Takeaway, Here & Now, Your Call, Radio Times, Sounds Good, St. Louis on the Air), Prehospital and Retrieval Medicine Podcast, Slate, Emergency Medicine News, MGH Charged Podcast, Side Effects, JAMA Medical News Summary	United States, Australia
	Press release	10 (3)	Moms Demand Action, Everytown, EurekAlert, Brady, Dutch Ruppersberger for Congress, Temple Health, Congresswoman Robin Kelly, Brown University, Giffords, National Physicians Alliance	United States
	Petition	7 (2)	Civic Action, Every Action, Affirm Research, One Pulse for America, reddit, Society for Adolescent Health and Medicine, care2	United States
	Summary	3 (1)	Kaiser Health News, The Trace, Google News	United States
	Testimony	1	House.gov	United States
	Letter	1	Orange County Brady Campaign Chapter	United States
	Fundraiser	1	Custom Ink	United States
	Event	1	Raleigh	United States

^a^Select publishers/hosts listed

NEJM: New England Journal of Medicine, JAMA: Journal of the American Medical Association, BMJ: British Medical Journal, UCLA: University of California, Los Angeles, NPR: National Public Radio, MGH: Massachusetts General Hospital

### Scientific publications

A total of n = 15 items met study inclusion criteria out of n = 114 search results. The first article was published on November 12^th^, 2018 as a news commentary in the British Medical Journal [25]. The month with the most items published was December 2018 (n = 5, 33%). Ten of the fifteen scientific items had Altmetric scores, with a median score of 46 (IQR 15, 111). The highest score was 616 for a New England Journal of Medicine article published on January 31^st^, 2019 entitled, “#ThisIsOurLane–Firearm Safety as Health Care’s Highway” [26]. This score represents citations by seven news outlets, three blogs, 759 tweeters, four Facebook pages, and 98 Mendeley references, among others, and puts the article in the 99^th^ percentile for attention score compared to contemporary research outputs [26]. The next highest scores were 526 for an Annals of Internal Medicine article published December 18^th^, 2018 [27], and 117 for a January 15th, 2019 Journal of the American Medical Association article [28]. None of the scientific items were peer-reviewed studies, but rather opinion pieces, sponsored events, podcasts, petitions, graphics, or news articles (Table 2).

### Significant events

No definitive association was found between message volume and certain high-profile firearm violence and policy events, as depicted in Fig 1, yet there are some potential links. The item considered to have sparked the #ThisIsOurLane movement was a position paper from the ACP on reducing firearm injuries and deaths in the United States, e-published on October 30^th^, 2018 [4]. Since publication, this article has achieved an Altmetric score of 3099, placing it in top five percent of all research scored by Altmetric [4]. This document prompted the NRA to post a tweet on November 7^th^, 2018 telling “self-important anti-gun doctors to stay in their lane,” along with an article challenging the ACP position paper claims [5, 29]. At the time of publication, the NRA’s tweet had received 3100 likes, 1200 retweets, and over 20,000 comments [5]. Later on November 7^th^, a mass shooting in Thousand Oaks, California at the Borderline country music bar killed 13 people, some of whom had survived the Las Vegas shooting only a year prior [30].

In response to the large #ThisIsOurLane #ThisIsMyLane response on social media the Twitter account @ThisIsOurLane was created [21]. On November 14^th^, 2018 the American College of Surgeons Committee on Trauma Firearm Strategy Team Workgroup released their initial set of recommendations to reduce firearm injury, death, and disability in the United States [31]. Amidst multiple television and radio interviews with prominent physicians and researchers in the field, the November 14^th^ Dr. Oz Show featured select experts and leaders of the #ThisIsOurLane movement to discuss the firearm violence crisis in the United States (Fig 1) [32]. A domestic violence shooting incident on November 19^th^ at Mercy Hospital in Chicago, which killed a police officer, a pharmacist, and an Emergency Medicine physician, did show a subsequent message volume spike on Twitter and in the mass media [33]. A second increase in Twitter message volume occurred on November 29^th^, corresponding with a #ThisIsOurLane Press Conference on Capitol Hill hosted by Congresswoman Robin Kelly, an event organized in response to the *This Is Our Lane* movement that included healthcare professionals from around the country [34].

Building on the momentum generated by the *This Is Our Lane* movement, the American College of Surgeons Committee on Trauma hosted the inaugural Medical Summit on Firearm Injury Prevention on February 10^th^ and 11^th^, 2019, bringing together the American Bar Association and representatives from 43 professional medical and injury prevention groups. The focus was on inclusivity, collegiality, and a public-health approach, in order to arrive at apolitical, consensus-based solutions to the firearm crisis in the United States [35, 36]. House Bill HR8 Bipartisan Background Checks Act of 2019 for new background check requirements between private parties passed on February 27, 2019 by 240 yeas to 190 nays, and continues to await consideration by the Senate at the time of publication [37].

Regarding U.S. mass shootings, there were n = 86 events that met the criteria outlined by the Gun Violence Archive over the 19-week (133 day) study period [17], equating to one mass shooting every other day. We excluded these data in a figure due to an inability to reliably ascribe changes in message volume with these continually occurring firearm violence events.

## Discussion

This study highlights the unique role social media can play to accelerate healthcare advocacy movements over traditional mass media and scientific publications. Similar results have been seen when using social media to track infectious disease outbreaks. Data from informal media are available earlier, facilitating more timely estimates and planning in dynamic situations, while subsequently correlating with official reports, such as for the 2010 Haiti cholera outbreak [38] and the 2019 SARS-CoV-2/COVID-19 global pandemic [39, 40]. Our results also suggest that social media potentiates traditional methods of mass communication, with the possibility for multidirectional synergy and amplification between all media types, as well as prompting off-line organized activities. This is highlighted by the initial activity spike on social media followed by the other mediums, scientific publications with high Altmetric scores, mentions of the hashtags throughout mass media, and temporally downstream, related real-world events. Even with the strong promise of social media, diverse types of publications remain valuable due to social and professional norms for receiving information and sharing evidence, as well as differing data source strengths and impacts.

Reliably studying how media advocacy leads to real-world change in healthcare policy continues to be challenging due to the complex nature of the intervention, potential confounders, and the issues being impacted [41, 42]. A 2017 systematic review assessing how planned media interventions affect the health policy-making process found interventions usually had a positive impact, including initiating policy discussions, influencing policy formation and adoption, increasing policymaker awareness, and improving compliance with laws and regulations. The only identified negative effect was opponent mobilization of the targeted policy in one study on underage drinking policies in Louisiana, leading to defeat of the bills being advocated for. Study authors speculated that this could have been related to more effective counter-media messaging funded by influential special interests and stakeholders [12]. Yet few studies have looked into the longitudinal relationship between media content and policy output, and those who have typically find mixed results, necessitating more robust research [43].

Examples of social media advocacy in health are even fewer than mainstream media, with nearly nonexistent studies on spontaneous movements like *This Is Our Lane*. Research primarily consists of case studies like Hefler et al. [44] on the strategic use of a tobacco control Facebook group to stimulate protests of tobacco company-sponsored concerts in Indonesia. Additional examples include a Planned Parenthood social media campaign to reverse funding decreases [41] and the *Ni Una Menos* (not one less) movement against femicide in Argentina [45]. Freeman et al. [46] reviewed nine corporate and public health case studies of planned social media campaigns and highlighted lessons learnt, with some of the recognized practices occurring during the #ThisIsOurLane campaign despite its spontaneous nature. These strategies include tapping into existing networks to build online communities and using engaging content that has a clear call to action. However, the authors recognized the continued gap of correlating online engagement with real-world action and behavior change, and the need to pursue more outcome-based measures. Additional tactics are provided in a 2020 systematic review on effective dissemination strategies for research to U.S. policymakers [47]. While print materials and personal communication were the most frequently used communication channels, some studies also employed traditional and social media, with the most successful approach being to start early, use champions and brokers, understand the stakeholders and process, know the context, engage supporters, and ensure timeliness, relevance, and accessibility of the topics, aligning with many characteristics of the *This Is Our Lane* movement. However the authors note further studies are needed to investigate exactly how the various communication channels effectively influence policy.

Social media not only provides increased opportunities for engagement from its open access design and heightened messaging speed, but also enables more flexible content. A 2015 review of 105 advocacy organizations’ use of Twitter showed the most effective advocacy messages, as measured in retweets, used hashtags that targeted diverse interests and expressed either calls-to-action, public education, organization values and goals, or organization branding. Additionally, the most popular tweets were those that contained photos and were sent from active, well-established accounts with more followers [48], characteristics of many of the tweets in the *This Is Our Lane* movement.

The photos, videos, and personal stories shared on Twitter exhibited authentic voices in the community that used techniques emblematic of public narrative. One method at work was the “identifiable victim effect,” which refers to a personal representation of victims generating more empathy and personal sacrifice than impersonal statistics [49]. This effect is thought to have played a role in stimulating action in other public health crises via the use of photographs, such as the Flint, Michigan water emergency and the AIDS epidemic, among others [50]. Healthcare workers united in the #ThisIsOurLane #ThisIsMyLane community to create a thoughtful dialogue based on a threat to their collective identities: senseless, unpredictable injury and deaths due to firearm-related violence. Public narrative is a method of translating personal values into motivation to stimulate action, such as creating a social movement. To do this it is critical to tap into common values by appealing to emotions, helping to understand the “why” of the issue at hand to facilitate mindful action [7], all of which can be accelerated by social media.

It is also important to recognize that values and politics, along with evidence, drive policy decisions [51]. A 2019 systematic review assessed the evidence for using narratives to impact health policy [52]. Although none of the eighteen studies contained explicit evidence linking the narrative intervention to the outcome, study findings suggested narratives may stimulate policy inquiries through inspiration and empowerment, provide education and awareness to motivate policy discussions, and serve as tools for advocacy and lobbying to draft, approve, or implement policy. However, the authors also warned of certain downsides of narratives, including the potential for bias due to selective inclusion or omission of details or limited viewpoints, and too heavy of a reliance on narrative over scientific facts, all of which supports the use of validity standards and narratives founded on evidence.

Despite the above information, some may still question the clinician’s role as an advocate. Healthcare workers are widely viewed as credible sources of health-related information for the public and policymakers alike [53]. Many are interested in reducing injustices in health, following in the footsteps of prior healthcare advocates who spoke up for water and sanitation systems, improved working conditions, criminal justice reforms, safe driving practices, and tobacco control, among others [54–56]. Furthermore, multiple professional organizations have released policy statements recognizing firearm-related violence as a public health crisis that warrants the involvement of health professionals [57]. A 2014 survey of state legislators highlighted important factors affecting whether health issues are placed on a policy agenda, with constituent needs and opinions, and scientific effectiveness of the intervention at the top of the list. These findings argue the need for informed citizens, such as healthcare professionals, to communicate with, educate, and mobilize constituents and stakeholders to advance critical public health policy, such as via social and traditional media [58]. Healthcare providers caring for firearm-injured patients have long understood the urgency to combat this public health crisis for the sake of their patients and loved ones. Via digital media using #ThisIsOurLane #ThisIsMyLane, healthcare professionals posted vivid photos and personal stories to form a community based on the shared values of an equal right to life and health. Subsequent organized action included a Capitol Hill press conference organized by Robin Kelley and petitions to Congress for increased research funding and background checks, both of which were passed by the House in Spring 2019 [37, 59]. Although a direct causal link cannot be made between the *This Is Our Lane* advocacy movement and these select policy successes based on available evidence, there are likely associations that should be further explored.

As we grapple with next steps for continued social media advocacy around healthcare-related issues like firearm violence, it is important to recognize that this method of communication is not innate but something many of us must learn. There are a variety of ways to become proficient at effective social media engagement, public narrative, and advocacy message framing, including professional society tutorials and events [60], published guides and consensus statements [61], and course work [62, 63], among others. These skills can be applied to interactions with elected officials, leveraging the respect of the medical profession to advocate and speak for those whose voices are not or cannot be heard. Interested clinicians should also explore professional society firearm-related activities to identify opportunities for expansion or collaboration, as well as to initiate multidisciplinary, multisectoral discussions on firearm violence with a public health approach. It is also critical to be aware of how public health issues are framed in the media, and actively move away from episodic or portrait stories to more thematic, landscape viewpoints. This perspective shift transfers the responsibility from the individual to the community, more accurately describing how an event occurred in the context of its surroundings and making the public health problems more visible [56]. There is also a need to further study the effects of media advocacy and public narrative on the actual adoption of public and health policy, such as via qualitative approaches, discourse analysis, and realist evaluation studies, which focus on the implementation contexts and processes that yield impact versus the more traditional intervention and outcome [12, 52].

Limitations of this study include the short timeframe due to limited access to Symplur data. However, as seen through our analyses, the most active time points were captured with subsequent lower publication quantities across all media types at later dates during the study period. Symplur relies on hashtag registration to ensure accurate metrics, and also has limits on the amount of retrospective data they can receive from Twitter, with potentially not all tweets for the two hashtags being captured prior to their registration. Due to Symplur not routinely tracking accounts, in-depth analytics were not available for the @ThisIsOurLane handle. Additionally, the Symplur data only included the hashtags’ use on Twitter, and did not quantify posts on other platforms nor related posts on Twitter that did not use or misspelled the hashtags. Symplur is also unable to filter out uses of the hashtag unrelated to firearms. Demographic data provided by Symplur is restricted to what users share in their profiles, and default healthcare stakeholder categories do not enable access to more granular data besides doctor or healthcare professional. Therefore we were unable to account for specific healthcare professional participants, such as nurses, advanced practice providers, etc. However, none of the above is thought to significantly alter our conclusions. Finally, mass media searches may not have located all forms equally, especially television and radio spots. To combat this potential source of bias, a diverse array of search engines and terms were used. Additionally, library news databases appeared to list many non-print media by their transcripts, facilitating inclusion of television and radio programs. The authors’ selection of events significant to the movement could have potentially missed important occurrences that only slightly influenced message or publication volume, or provided additional evidence of off-line events in response to the movement. Yet we feel the risk is low given the collective expertise and involvement of the authors in the movement’s pivotal events, as well as a lack of unexplained message volume spikes.

## Conclusions

Firearm injuries and deaths remain a critical issue in the United States. Healthcare providers are in a unique position to witness and treat the devastating aftermath, while also understanding the root causes that can prevent the injuries from occurring in the first place. Through the power of social media and public narrative, medical professionals can quickly transform impersonal, sterile statistics into real people through authentic storytelling and serve as compelling advocates for issues affecting population health. These stories can link us through shared values and experience, and unite us toward a common task --- to address firearm violence and other health threats with a public health approach, and identify concrete interventions that protect individual liberties while giving everyone the opportunity to thrive.
